# Comparison of Life Traits in Two Bacterivorous Nematodes Suggest Different Ecological Strategies to Exploit Similar Habitats

**DOI:** 10.3390/life12101516

**Published:** 2022-09-28

**Authors:** Je-Hyun Moon, Rocel Amor Indong, Alfredo V. Alcantara, Kyoung-hye Yoon, Jin I. Lee

**Affiliations:** 1 Division of Biological Science and Technology, College of Science and Technology, Yonsei University, Mirae Campus 304, 1 Yonseidae-gil, Wonju 26493, Gangwon-do, Korea; 2 Department of Physiology, Mitohormesis Research Center, Wonju College of Medicine, Yonsei University, Wonju 26426, Gangwon-do, Korea

**Keywords:** nematode, *C. elegans*, *Acrobeloides*, soil ecology, fertility, lifespan

## Abstract

Environments can be in states of dynamic change as well as persistent stability. These different states are a result of outside external conditions, but also the constant flux of living organisms in that ecological fauna. Nematodes are tremendously diverse, and many types can reside in the same soil microenvironments at the same time. To examine how so many nematodes can thrive and exploit a single environment, we identified two bacterivorous nematodes, *Caenorhabditis elegans* and *Acrobeloides tricornis*, that can inhabit rotting apple and soil environments. We cultured both nematodes in the laboratory and compared their life traits. We found that whereas *C. elegans* develops and reproduces extremely quickly, *A. tricornis* reaches sexual maturity much later and lays eggs at a slower rate but remains fertile for a longer time. In addition, *A. tricornis* displays a slower feeding behavior than *C. elegans*. Finally, *A. tricornis* has a significantly longer lifespan than *C. elegans*. These differences in development, physiology and behavior between the two nematodes hint at different ecological strategies to exploit the same habitat over different time periods, *C. elegans* as a colonizer-type nematode, and *A. tricornis* as more of a persister.

## 1. Introduction

The biological and physiological design of animal species are elegantly adapted to the surrounding environment in order to increase their reproductive fitness and maintain their evolutionary struggle. This is particularly true for adaptations such as fertility and longevity, in which some environments favor reproductively fast animals and others benefit persistence and animals with a longer lifespan.

Nematodes, which may account for 80% of all individual animals on the earth, are tremendously diverse with possibly over one million species inhabiting nearly every ecosystem on earth [1,2]. However, individual species of nematodes have specific physiological characteristics that allow them to reside and thrive in particular fauna. This is best exemplified in soil ecology and the various nematodes that inhabit diverse soil faunas. Bongers [3] established a scale termed the “maturity index”, in which the type of nematodes inhabiting the soil could indicate the current ecological state of the soil environment. In particular, “colonizer” nematodes, characterized by short life cycles, high rates of reproduction, and a dauer stage, inhabit soils that are dynamic and could be either favorable or toxic [3,4,5]. On the other side of the spectrum “persister” nematodes are generally long lived with slow reproduction, and dwell in habitats characterized by long durational stability [3,4,5]. Soil environments will evolve over time, changing from a more ecologically dynamic and active environment favorable for colonizers to a more stable environment that supports persisters [4,5,6].

Studies in the laboratory have given some insight into comparative life traits for some bacterivorous nematodes such as Rhabditidae colonizer nematodes and Cephalobidae nematodes. For instance, although four species of Rhabditidae and three species of Cephalobidae nematodes showed varying brood sizes, the development and egg production period of all Cephalobidae nematodes was much longer than Rhabditidae [7]. In addition, the population growth of two Cephalobidae nematodes were more than four times slower than a Rhabtididae nematode [8].

The most well-characterized nematode is the Rhabtididae nematode *Caenorhabditis elegans*. First studied in the laboratory using genetics in the 1970s by Sydney Brenner [9], the development, physiology and behavior of the hermaphrodite *C. elegans* has been characterized exhaustively in the laboratory over the last 50 years. However, much of what we know about the natural ecology of *C. elegans* was not known until more recently. *C. elegans* can be found in diverse soil environments globally but usually in a dauer, non-proliferative form [10]. However, they can be found proliferating abundantly in dynamic environments such as soils with decaying vegetative matter and rotting fruits [11,12,13]. In particular, *C. elegans* can be found in farms and fruit orchards associated with rotting apples and other fruits [11,13,14], often reproducing to extremely high numbers in highly rotten apples. These soil environments associated with *C. elegans* confirm that they are colonizer nematodes and considered cp-1 on the Bongers 1 to 5 Maturity Index scale, just as other Rhabditidae nematodes [4,5].

Enriched soil environments such as those containing rotting fruit and vegetation can support all types of nematodes including many colonizers as well as some nematodes that are higher on the c-p maturity index scale. As those environments evolve over time from an ecologically early dynamic state to a later stable state, the populations of nematodes are thought to evolve together to consist of more persister-type nematodes [6].

In this study, we sought to collect nematodes that are associated with *C. elegans* favorable environments by rotting apples in highly organic peat soil outside. Among the samples collected we identified the Cephalobidae nematode *Acrobeloides tricornis*, previously identified in South Korea [15], that grew and reproduced in this environment. As all Cephalobidae nematodes are considered cp-2 on the Maturity Index scale, *A. tricornis* is likely more of a persister-type nematode than *C. elegans*. We wondered whether differences in biology and life traits could account for the varied ecological strategies for the two nematodes to exploit the same habitat. In the laboratory, we compared *C. elegans* and *A. tricornis* growth, fertility, lifespan and behavior together and show distinct differences between colonizer and persister nematode life traits.

## 2. Materials and Methods

### 2.1. Nematode Culture and Strains

*C. elegans* and *A. tricornis* nematodes were cultured on Nematode Growth Medium (NGM) agar seeded with *Escherichia coli* (*E. coli)* OP50 strain as previously described by Brenner [9]. *C. elegans* N2 Bristol strain was obtained from the *Caenorhabditis* Genetic Center (CGC) in University of Minnesota, USA, and *A. tricornis* isolation is described in the following cultivation and isolation protocol. Both strains were maintained in an incubator (JSRI-250C, JSR, Gongju, South Korea) at 20 °C.

### 2.2. A. tricornis Cultivation and Isolation

**Figure 1:**
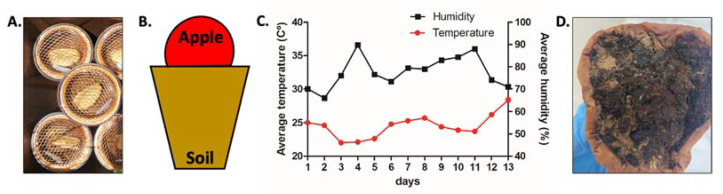
Experimental set-up to obtain wild nematodes from a rotting apple/soil environment: (**A**) apples placed on a peat soil-filled pot placed outdoors; (**B**) schematic of the apple/soil ecological environment; (**C**) average temperature (°C) and humidity (%) for the Yonsei University campus in Wonju, South Korea for 1 to 13 July 2021; (**D**) after over 24 days, nematodes from inside the open surface of the rotting apple and the adjacent soil were collected.

Commercially grown apples were cut in half and placed rind side up in a pot (130 mm) containing peat moss soil (Naro Farm) (Figure 1A,B). The pots were placed outside of the greenhouse located next to the Division of Biological Science and Technology building, at Yonsei University in Wonju, Gangwon Province, South Korea, in July 2021. The area where the pots were placed was partially shaded and vegetated. Each pot was watered with approximately 400 mL of water on non-rainy days to prevent drying of the soil. Temperature and humidity were tracked and recorded (Figure 1C) using the Korean National Weather Service website (https://www.weather.go.kr (accessed on 7 January 2021)). After 13 days, the pots were brought indoors where environmental conditions were more stable for another 11 to 13 days to allow the nematodes to grow and populate the sample. The temperature and relative humidity (RH) inside the room was measured using Testo 175H1 data logger (Seoul, Korea). The isolation of bacterivorous nematodes was performed using the plating method. This was done by cutting the rotten apple 2 cm thick from the exposed surface, then cutting this piece into several pieces, the pieces were then placed around the edge of an NGM plate containing a small OP50 strain *E. coli* lawn at the center. The same plating method was done using soil samples, where 2 cm of soil including the area that directly touched the rotten apple was plated (Figure 1D). Nematodes that came out from the rotten apple and soil were picked using the tip of a platinum wire and transferred to an empty NGM plate for maintenance.

### 2.3. Sequencing Analysis

Extraction of gDNA from *C. elegans* and *A. tricornis*, was conducted using single worm lysis method [16]. A single nematode was picked and transferred to a non-seeded NGM plate, then allowed to move freely for at least 10 min to remove bacteria on the body. The single worm was transferred to a PCR tube containing worm lysis buffer (90 μL 1 × PCR buffer + 5 μL 20mg/mL proteinase K) and the tube was frozen in −80 °C in a freezer for at least 5 min. The tube was then placed in a PCR machine (A-2040-1, Bioneer, Daejeon, South Korea) according to a single worm lysis program (60 min at 60 °C then 15 min at 95 °C to inactivate proteinase K). Nematode-specific primers for 18S ribosomal RNA (small subunit) gene[SSU18A (5′-AAAGATTAAGCCATGCATG)/SSU26R (5′-CATTCTTGGCAAATGCTTTCG), RHAB1350F (5′-TACAATGGAAGGCAGCAGGC)/RHAB1868R (5′-CCTCTGACTTTCGTTCTTGATTAA)] and the mtDNA cox-1 gene[Cepha_CO1_F(5′-ATGATTTTTTTTATGGTGATGCC)/Cepha_CO1_R(5′-ACTACAAAATATGTGTCATG)] [15,17,18] were used for sequencing after a general PCR with TaKaRa Taq^TM^.

### 2.4. Lifespan, Egg Laying Assay, Brood Size, and Body Length Measurement

Lifespan analysis was performed using synchronized worms. The synchronization of worm growth was done by allowing 10 adult nematodes to lay eggs on NGM agar for 6 h and then the adults were removed, and the eggs were incubated at 20 °C until hatching. Once these worms had at least one egg in their body, they were considered 1-day adults. Progenies from 1-day worms were distinguished by transferring gravid nematodes to new NGM plates every 2–3 days depending on how many offspring hatched. Survival was checked every day until all animal died, and dead worms were removed from the NGM plates. Brood size was measured by counting eggs every day. Body length was quantified by imaging day-1 worms using an Olympus BX50 microscope (Tokyo, Japan) equipped with Olympus DP74 camera (Tokyo, Japan), and measuring the length of each nematode using ImageJ software.

### 2.5. Measuring Fluorescence Accumulation

Ten adult nematodes were allowed to lay eggs on NGM agar seeded with *Enterobacter cloacae* (*E. cloacae*) *CEN2ent1*-tdTomato strain [19] for 6 h. *E. cloacae* was grown by inoculating 1 CFU of *CEN2ent1*-tdTomato strain in LB broth media with 100 μg/mL kanamycin and then incubating overnight at 37 °C, while shaking. Bacterial seeding of NGM plates was performed by placing 0.5 mL of *E. cloacae* inoculated LB broth media on the center of the NGM agar.

Afterwards, 1-day adult nematodes were collected in a 1.5 mL microcentrifuge tube and washed with S-basal buffer three times. Washed worms were anesthetized with 2 mM levamisole in S-basal then mounted on 2% (*w*/*v*) agar pads. Colonization was observed by taking fluorescent images using Olympus BX50 microscope equipped with Olympus DP74 camera and quantified by measuring the intestinal signal intensity using the ImageJ software.

### 2.6. Pharyngeal Pumping

Synchronized animals were placed on OP50 seeded NGM plate for 10min and allowed to freely move. Pharyngeal pumping was counted for 1 min by observation using a stereo microscope (SMZ18, Nikon, Tokyo, Japan). Pharyngeal pump was defined as grinder movement in the terminal bulb of the nematode’s head.

## 3. Results

After exposing our soil and apple sample to natural conditions outside, and allowing nematodes to populate the sample, we collected nematodes from the rotting apple and adjoining soil. From these samples we occasionally found different types of nematodes coming out from both the soil and apple. Among these we found one type of nematode in multiple samples that also grew and maintained well on the agar plates. In order to determine the identity, we sequenced areas of both the 18S rDNA gene and the *cox-1* gene and found that this nematode was identical in sequence to *Acrobeloides tricornis*. *A. tricornis* is found globally in diverse habitats [20,21,22,23] including South Korea [15], and can grow concurrently with *C. elegans* among other nematodes for weeks experimentally in decomposing soil habitats [24,25].

Whereas *C. elegans* as a cp-1 colonizer-type nematode displays fast development, short life cycle and high brood size, *Acrobeloides* nematodes are known to have slower development and population growth in comparison to *C. elegans* [7,8]. We wondered whether the life traits of *A. tricornis*, which can populate *C. elegans* favorable environments, suggests more of a colonizer or a persister-type nematode when compared with *C. elegans*. In order to investigate how *C. elegans* and *A. tricornis* can exploit and populate the same environments, we compared multiple life traits of the two nematodes.

**Figure 2:**
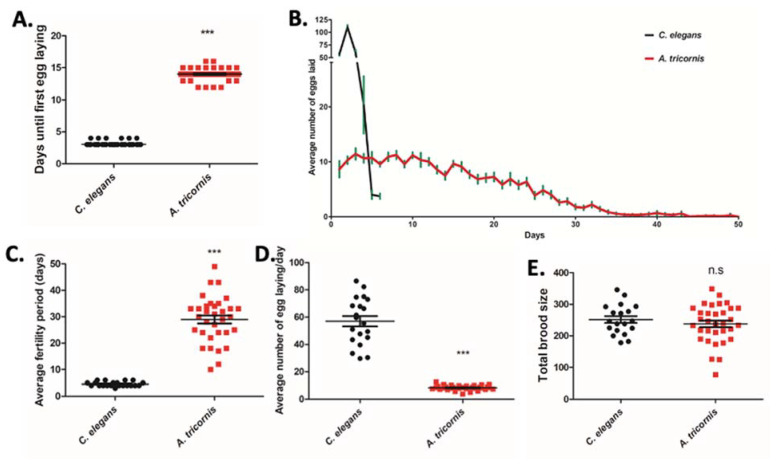
Comparison of fertility and reproduction phenotypes in *C. elegans* and *A. tricornis*: (**A**) number of days required from embryo stage to first egg laying in *C. elegans* and *A. tricornis*. Significant differences relative to *C. elegans* are indicated by a triple asterisk (*p* < 0.0001) based on Studen’s *t* test. Error bars indicate mean ± s.e.m. n > 70 for both nematodes; (**B**) number of eggs laid on each day until worms lose fertility. Error bars indicate mean ± s.e.m. At least n = 20 for all nematodes; (**C**) average fertility period for each nematode. Fertility period is defined as the number of days that each worm laid eggs from first egg laying. Significant differences relative to *C. elegans* are indicated by a triple asterisk (*p* < 0.0001) based on Studen’s *t* test. Error bars indicate mean ± s.e.m. n > 20 for all nematodes; (**D**) quantification of the average number of eggs laid per day during the fertility period in denoted species. Significant differences relative to *C. elegans* are indicated by a triple asterisk (*p* < 0.0001) based on Studen’s *t* test. Error bars indicate mean ± s.e.m. n > 20 for all nematodes; (**E**) quantification of the total brood size of denoted species. n.s, not statistically significant (*p* > 0.05) based on Stuent’s *t* test. Error bars indicate mean ± s.e.m. n > 20 for all nematodes. All the experiments were repeated at least three times.

*A. tricornis*, like *C. elegans*, can grow and populate on Nematode Growth Medium (NGM) agar seeded with *E. coli* bacteria. The hermaphrodite *C. elegans* reproductive life cycle, the time from egg to sexually-mature adulthood, is a short 72 h, whereas *A. tricornis*, which likely reproduces parthenogenetically [15,26], begins to lay eggs 336 h, 2 weeks after they themselves were laid (Figure 2A). After egg-laying in *C. elegans* commences, the hermaphrodite mothers lay large numbers of eggs for 3 days, averaging about 56.96 eggs per day during those days (Figure 2B–D). In contrast, *A. tricornis* continues to lay eggs for almost 29 days, averaging only 8.357 eggs per day (Figure 2B–D). Although the timing of reproduction and the rate of reproduction in *C. elegans* and *A. tricornis* are vastly different, the overall brood size of the nematodes, the total number of eggs laid over a lifetime, is similar, 251.6 for *C. elegans* and 238.0 for *A. tricornis* (Figure 2E).

**Figure 3:**
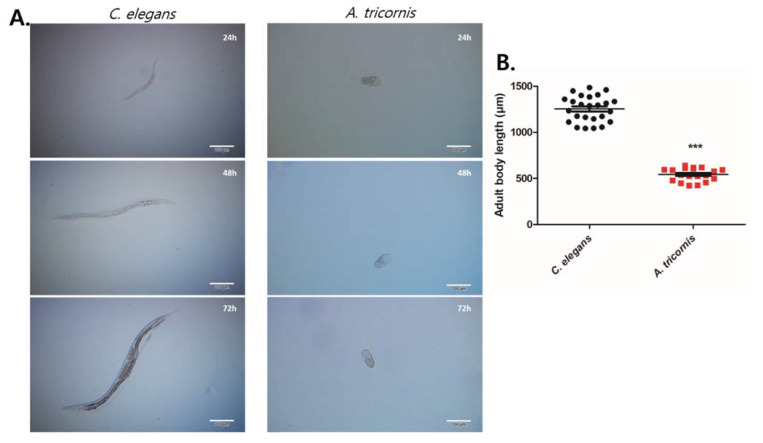
Differences in growth and development between *C. elegans* and *A. tricornis*: (**A**) images of developmental stage of *C. elegans* (**right**) and *A. tricornis* (**left**) from embryo for every 24 h until 3 days. Scale bar represents 200 µm for right panels and 100 µm for left panels; (**B**) quantification of adult worm body length of denoted species. Significant differences relative to *C. elegans* are indicated by a triple asterisk (*p* < 0.0001) based on Stuent’s *t* test. Error bars indicate mean ± s.e.m. n > 19 for all nematodes. The experiments were repeated at least three times.

*C. elegans* and *A. tricornis* also grow and develop at greatly different rates. After 72 h of being laid, the *C. elegans* embryo grows into a mature adult, whereas *A. tricornis* has not even hatched from the egg yet (Figure 3A). There is also a large difference in the size of the fully grown adult—*C. elegans* has an average body length of 1254 µm and *A. tricornis*, 543 µm (Figure 3B).

The pumping behavior of the pharynx allows bacterivorous nematodes to draw in bacteria, grind their food, and pump both live and dead bacteria into their intestines. 

**Figure 4:**
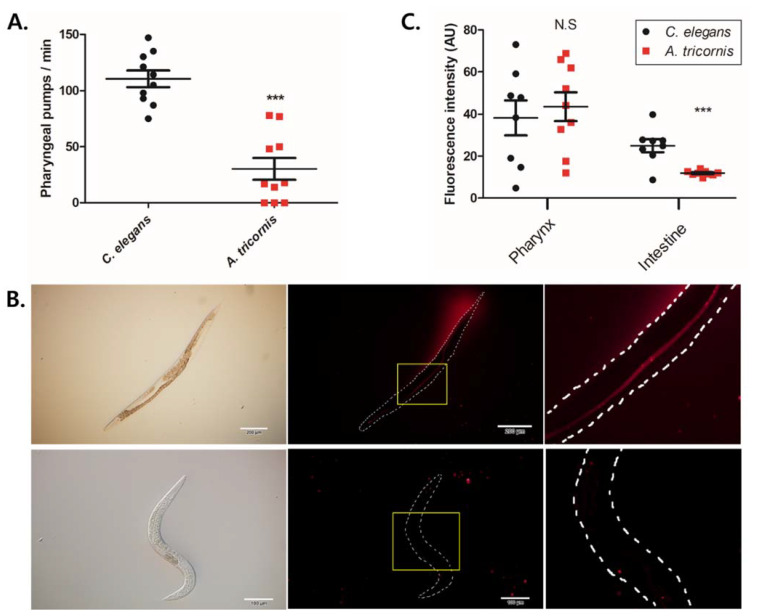
Comparison of pharyngeal pumping behavior and internal uptake of bacteria: (**A**) quantification of pharyngeal pumps per min of *Caenorhabditis elegans* and *Acrobeloides tricornis*. Significant differences relative to *C. elegans* are indicated by a triple asterisk (*p* < 0.0001) based on Stuent’s *t* test. Error bars indicate mean ± s.e.m. At least n = 10 for all species; (**B**) fluorescent images of accumulated tdTomato-labeled *E. cloacae* bacteria in *Caenorhabditis elegans* (**top**) and Acrobeloides tricornis (**bottom**) after 7 h of feeding. The images of the inset (**left**) are zoomed-in views of the region indicated by the yellow boxes. Scale bar represents 200 µm for top panels and 100 µm for bottom panels; (**C**) quantification of fluorescence intensity of pharynx and intestine accumulated tdTomato-labeled *E. cloacae* bacteria in denoted species. Significant differences relative to *C. elegans* are indicated by a triple asterisk (*p* < 0.0001) based on Stuent’s *t* test. Error bars indicate mean ± s.e.m.; n.s, not statistically significant (*p* > 0.05). n > 8 for all species. All the experiments were repeated at least three times.

As this has direct implications in growth body size [27,28], we measured and compared the pharyngeal pumping rate of *C. elegans* and *A. tricornis*, and found that *A. tricornis* displays an average of only 30.2 pumps per minute, about five times slower than *C. elegans*, which has an average of 110.5 pumping rate (Figure 4A).

We wondered whether this may affect the uptake of bacteria into the intestine of *A. tricornis*. We incubated the nematodes together with a fluorescent tdTomato-labeled *E. cloacae* bacteria that *C. elegans* can readily consume, and whose fluorescence can be observed in the pharynx and the intestinal lumen [19]. We found that, like *C. elegans*, *A. tricornis* nematodes display accumulation of fluorescent bacteria in their pharynx (Figure 4B,C). However, unlike *C. elegans*, the *E. cloacae* bacteria does not accumulate in the *A. tricornis* intestine (Figure 4B,C).

**Figure 5:**
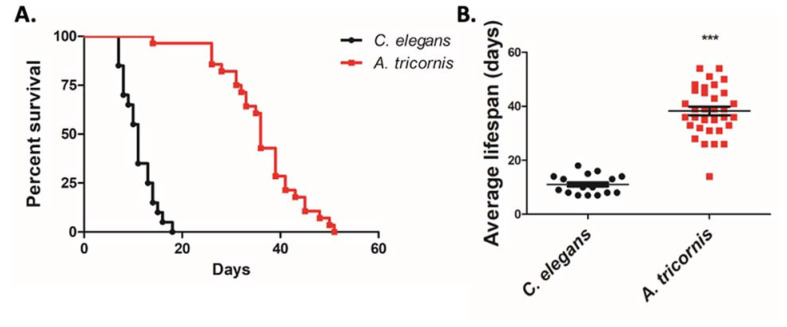
Differences in lifespan of *C. elegans* and *A. tricornis*: (**A**) Kaplan-Meier curves from lifespan assay for *Caenorhabditis elegans* and *Acrobeloides tricornis*. Statistical tests for significant difference between survival curves were performed using the log-rank test (*p* < 0.0001); (**B**) quantification of average lifespan of denoted species. Significant differences relative to *C. elegans* are indicated by a triple asterisk (*p* < 0.0001) based on Stuent’s *t* test. Error bars indicate mean ± s.e.m. n > 20 for all species. The experiments were repeated at least three times.

Finally, we compared the survival and lifespan of the two nematodes from adulthood until death. We found that 50% of *C. elegans* were dead within 11 days with an average lifespan of 11.05 days (Figure 5A,B), similar to other reports [29]. In contrast, 50% of *A. tricornis* remained alive at 36 days, with an average lifespan of 38.27 days, almost four times longer than *C. elegans* (Figure 5A,B). A portion of *C. elegans* died due to internal hatching of embryos, whereas none of the *A. tricornis* mothers died due to internal hatching.

## 4. Discussion

Related animals may occupy the same spatial ecological niche, but how these animals grow and populate an evolving environment may diverge based on inherent physiological differences in development, fertility, and lifespan. *C. elegans* is known to quickly and profusely populate rotting apples on the ground of an orchard [11,13,14]. Although *C. elegans* has never been isolated in nature in South Korea and rarely in East Asia [30], we sought to isolate other nematodes in South Korea that could also populate a similar apple environment. From this experiment we found that *A. tricornis* thrived in this environment.

In this study, we measured several aspects of *A. tricornis* development, physiology, and behavior in laboratory culture (Table 1). Although at least one other group has grown *A. tricornis* in laboratory conditions on agar plates seeded with *E. coli* bacteria [31], this is the first report of *A. tricornis* growth, fertility, lifespan and pharyngeal pumping behavior that we know of. The isolation and characterization of a single female *A. tricornis* by another group in South Korea showed a shorter body length than what we report [15], however different conditions of growth in the laboratory and in nature may account for this discrepancy. Compared to laboratory culture of another *Acrobeloides* nematode, *A. nanus*, we find similar timing of development, and a similar body length, but differences in the period of fertility, brood size and lifespan [32]. This discrepancy may be due to the different species but may also be due to differences in culture conditions.

We compared the two nematodes and found large variations in the timing of development, fertility, behavior, and lifespan (Table 1). For instance, *C. elegans* finishes laying all of its eggs one week before *A. tricornis* even starts to lay eggs. Thus, several generations of *C. elegans* will boom in population before *A. tricornis* has reached sexually maturity. Others have also grown *C. elegans* and *A. tricornis* and other *Acrobeloides* nematodes and have noted the early burst in population of *C. elegans* compared to *Acrobeloides* [7,8,25,33]. The faster pharyngeal pumping rate and increased bacterial load in *C. elegans* intestine hints at a heightened ability to take advantage and rapidly consume abundant bacterial food. In contrast, *A. tricornis* displays a much longer fertility period and lifespan than *C. elegans* suggesting a capability for *Acrobeloides* nematodes to persist reproductively for long periods in a soil ecosystem. These genetically-fixed phenotypes in *C. elegans* and *A. tricornis* nematodes define their designation as colonizer and persister nematodes, respectively, even when they dwell together in the same habitat.

How can these two nematodes coexist in the same ecological niche? This seems difficult given *C. elegans* ability to quickly populate and drain bacterial resources before *Acrobeloides* nematodes even reproduce. However, our study does not address several factors that would exist in nature. First, natural environments our comprised of diverse bacteria and nematodes themselves have preferred food sources, consuming some types of bacteria and leaving others [33]. Bacteria that *C. elegans* does not consume well may be left behind for *A. tricornis* to eat. Second, *C. elegans* and *Acrobeloides* have different resistance to environmental conditions such as temperature. *C. elegans* grows and reproduces extremely well at 20 °C but cannot survive or reproduce at temperatures above 30 °C, which is the temperature that *A. bodenheimeri* and *A. buetschlii* thrive in [8]. At such high temperatures, as well as in crowded conditions, *C. elegans* larvae will enter a dauer stage, attempting to escape the unpleasant conditions while leaving other nematodes exploit the environment. Finally, nematodes can move to other areas within the soil ecosystem to search for a more optimal environment. As ecological succession of a soil environment progresses over time, some nematodes will populate deeper or shallow areas over that time frame [6]. Furthermore, in single rotting apples found in an orchard that contained both *C. elegans* and *C. briggsae* nematodes, *C. elegans* generally populated the bottom portion of the apple which was not observed in *C. briggsae* [13]. This supports the notion that different types of nematodes can populate separate microhabitats in the same ecological environment.

The fact that the cp-1 nematode *C. elegans* and the cp-2 nematode *A. tricornis* are found in the same enriched soil environment with rotting apples is supported by the idea of nematode functional guilds previously described that further accounts for nematode life traits to characterize soil food webs [34]. The authors here describe soil ecological environments as either basal, soils that are ecologically simple or limited in resource; as structured, soils that have more ecological complexity and more resources; or as enriched, soils that were recently flush with microbial resources. Our environment described in Figure 1, which includes an apple and a commercially available peat soil, is most similar to a basal enriched environment. According to Ferris et al., this type of environment would include a guild of nematodes comprised of cp-2 nematodes that populate basal soils, and cp-1 nematodes that can quickly exploit sudden blooms of bacteria in enriched soils [34]. Thus, the life traits we describe here for the two different nematodes that both inhabit this specific ecological environment supports this idea of the functional guilds.

Although *C. elegans* exploits and multiplies to enormous numbers in rotting apples within days [14], we made observations that *A. tricornis* can proliferate slowly over 3 to 4 weeks to the thousands in our rotting apple experiment after 3–4 weeks. It would be interesting in the future to co-culture *C. elegans* and *A. tricornis* in rotting apple and soil in controlled conditions to observe how the two nematodes populate a single ecological habitat.

## 5. Conclusions

Although *C. elegans* and *A. tricornis* can both reside in an enriched soil and rotting apple habitat, differences in the timing of their development, fertility, and lifespan, as well as their feeding behaviors, may explain how the two bacterivorous nematodes can exploit and populate the same environment.

## Figures and Tables

**Table 1:** Comparison of life cycle parameters of *Caenorhabditis elegans* and *Acrobeloides tricornis*.

	Body Length (µm)	Growth Duration from Egg to Adult (Days)	Fertility Period (Days)	Eggs Laid/Day	Total Brood Size	Pharyngeal Pump/min	Lifespan (Days)
*C. elegans*	1254 ± 28.04	3.04 ± 0.018	4.57 ± 0.190	56.96 ± 3.838	251.6 ± 10.86	110.5 ± 7.258	11.05 ± 0.720
*A. tricornis*	543.0 ± 16.29	14.03 ± 0.085	28.97 ± 1.538	8.36 ± 0.296	238.0 ± 10.60	30.20 ± 9.721	38.27 ± 1.581

All measurements are represented in the form: mean ± s.e.m.

## Data Availability

Data, resources and reagents will be made available, and requests should be directed to and will be fulfilled by the lead contact Jin I. Lee (jinillee@yonsei.ac.kr).
